# The contribution of the CRP/CD64 axis to renal cancer progression by inducing protumor activation of tumor‐associated macrophages

**DOI:** 10.1002/cti2.70013

**Published:** 2024-11-19

**Authors:** Cheng Pan, Yukio Fujiwara, Hiromu Yano, Toshiki Anami, Yuki Ibe, Lianbo Li, Yuji Miura, Takanobu Motoshima, Shigeyuki Esumi, Junji Yatsuda, Taizo Hibi, Tomomi Kamba, Yoshihiro Komohara

**Affiliations:** ^1^ Department of Cell Pathology, Graduate School of Medical Sciences Kumamoto University Kumamoto Japan; ^2^ Department of Urology, Graduate School of Medical Sciences Kumamoto University Kumamoto Japan; ^3^ Department of Pediatric Surgery and Transplantation, Graduate School of Medical Sciences Kumamoto University Kumamoto Japan; ^4^ Department of Medical Oncology Toranomon Hospital Tokyo Japan; ^5^ Department of Anatomy and Neurobiology, Graduate School of Medical Sciences Kumamoto University Kumamoto Japan; ^6^ Center for Metabolic Regulation of Healthy Aging Kumamoto University Kumamoto Japan

**Keywords:** CD64, CRP, IL6, macrophage, PD‐L1, renal cell carcinoma

## Abstract

**Objectives:**

C‐reactive protein (CRP) is a well‐known acute‐phase protein that increases remarkably under various inflammatory conditions and is elevated in patients with malignant tumors. In this study, we investigated the influence of CRP on the tumor microenvironment in clear cell renal cell carcinoma (ccRCC).

**Methods:**

This study explored CRP's role in ccRCC by co‐culturing human macrophages with ccRCC cells and employing antibody blocking, RNA sequencing and *in vitro* experiments for functional insights. We also analysed The Cancer Genome Atlas Program (TCGA) data to link CD64 expression with ccRCC prognosis and used immunohistochemistry to associate CD64^+^ macrophages with tumor severity and systemic CRP levels.

**Results:**

A co‐culture study using human macrophages and RCC cell lines showed that CRP‐stimulated macrophages secrete IL‐6, which induces RCC proliferation via STAT3 activation. CRP‐induced protumor activation of macrophages was suppressed by CD64 blocking antibodies. Furthermore, CRP elevates PD‐L1 expression in macrophages via the CD64‐STAT1 signalling pathway. Statistical analysis of TCGA data indicated that increased CD64 expression was associated with a worse clinical course in ccRCC. Immunohistochemical analysis of pathological specimens revealed that high CD64 expression in tumor‐associated macrophages (TAMs), and a high density of CD64^+^ TAMs, was linked to high nuclear grade and stage. High CD64 expression was also correlated with increased serum CRP levels.

**Conclusions:**

The CRP‐CD64 signal was linked to the protumor activation of TAMs and could be a promising target for anticancer immunotherapy in ccRCC.

## Graphical abstract

In this study, we found that C‐reactive protein (CRP) activates protumor functions of tumor‐associated macrophages. This study suggests that the CRP‐CD64 signal could be a promising target for anticancer immunotherapy.

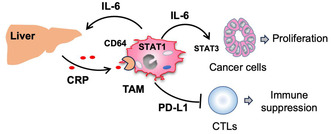

## Introduction

Acute‐phase proteins are produced from hepatocytes by immune cell‐derived cytokines, such as IL‐6 and TNF‐α. Increased blood concentrations of these proteins have been observed in patients with inflammatory diseases and malignant tumors.^1^ C‐reactive protein (CRP) is an acute‐phase protein that is well‐known for its clinical role as an indicator of inflammation or infection. The serum CRP level is remarkably increased by various inflammatory and infectious conditions, but changes in a low level equally quickly after the removal of the pathological condition.^2^, ^3^ Various factors, such as IL‐6 and IL‐1β, induce the production of CRP from hepatocytes by activating transcription factors, including C/EBP, STAT3, c‐Fos and NF‐κB, in the acute phase. Phosphorylcholine (PCh), C1q and Fcγ receptors (e.g. CD64) are known to be specific binding molecules for CRP; their interactions play important roles in innate immunity. CRP also activates the classical complement pathway by binding to C1q. The binding of Fcγ receptors to CRP also induces the secretion of pro‐inflammatory cytokines in phagocytes.^2^, ^3^, ^4^

In a large number of studies focussing on various tumors, serum CRP has been identified as a factor that predicts prognosis, tumor recurrence and treatment responses.^5^, ^6^ Several studies have revealed that CRP is detected in clear cell renal cell carcinoma (ccRCC) tissues and that there is a positive correlation between CRP expression in tumor tissues and poor prognosis in patients.^7^, ^8^ However, the role of CRP in tumor progression remains unknown. Furthermore, several studies using human tumor tissues have shown that serum CRP levels are correlated with the formation of an immunosuppressive microenvironment in hepatocellular carcinoma and ccRCC.^9^, ^10^ Although they have overwhelmingly focussed on the role of CRP as a predictive marker for tumor prognosis, no studies have focussed on CRP‐induced tumor promotion and progression as a functional molecule.

Recent studies have indicated the significance of the tumor immune microenvironment (TIME) in tumor growth, progression, metastasis and immunosuppression. In cells constructed with the TIME, macrophages are the main components of RCC.^11^ Infiltrating macrophages in tumor tissues are called tumor‐associated macrophages (TAMs), which secrete various factors related to tumor cell growth, angiogenesis and immunosuppression.

In this study, to reveal the protumor functions of CRP in the TIME via the signalling of CRP receptors, such as CD16,^12^ CD32^13^ and CD64,^14^ and cytokine production from macrophages, we examined the effect of CRP on tumor progression via the functions of TAMs using human primary macrophages and pathological specimens of ccRCC.

## Results

### CRP‐treated macrophages promote RCC proliferation via the IL‐6/STAT3 pathway

**Figure 1:**
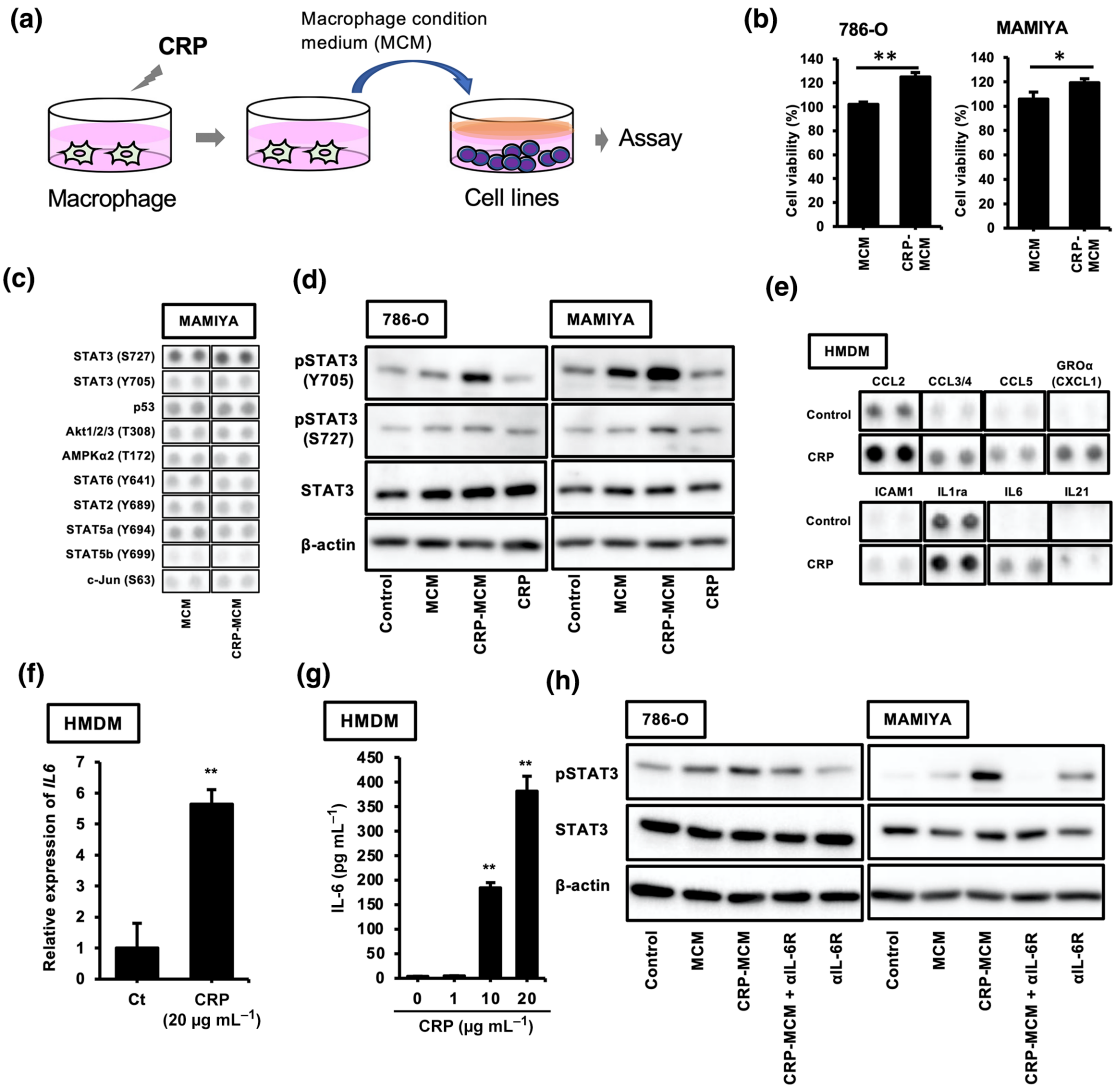
CRP‐treated macrophages promote tumor proliferation in ccRCC cell lines via STAT3 activation. **(a)** A schematic diagram of the CRP‐MCM stimulation experiments in ccRCC cell lines. **(b)** The proliferation of the ccRCC cell lines (786‐O and MAMIYA) stimulated with MCM or CRP‐MCM for 48 h was measured by WST‐8 assay (*n* = 6). The Mann–Whitney *U*‐test was performed. *P < 0.01, **P < 0.001. **(c)** The results of the kinase phosphorylation array for the measurement of kinase activity in MAMIYA cells stimulated with MCM or CRP‐MCM. **(d)** The phosphorylated STAT3 (Tyr 705 and Ser 727) expression in ccRCC cell lines (786‐O and MAMIYA) after treatment with MCM or CRP‐MCM for 24 h was measured by western blotting. Experiments were repeated three times with almost identical results. **(e)** The results of the cytokine array analysis for measuring cytokines contained in the culture supernatant of HMDMs stimulated with 20 μg mL^−1^ CRP. **(f)** The IL‐6 mRNA expression in HMDMs stimulated with 20 μg mL^−1^ CRP was determined by real‐time PCR (*n* = 3). Experiments were repeated three times with almost identical results. The Mann–Whitney *U*‐test was performed. *P < 0.01, **P < 0.001. **(g)** The IL‐6 secretion from HMDMs stimulated with 20 μg mL^−1^ CRP was determined by ELISA (*n* = 4). Experiments were repeated three times with almost identical results. The Kruskal–Wallis test was performed. *P < 0.01, **P < 0.001. **(h)** The phosphorylated STAT3 expression in ccRCC cell lines (786‐O and MAMIYA) after treatment with MCM or CRP‐MCM during incubation with αIL6R Ab (20 μg mL^−1^) for 24 h was measured by western blotting. Experiments were repeated three times with almost identical results.

A cell culture study using macrophages and RCC cell lines was performed to test whether CRP stimulation influenced the protumor functions of macrophages, as shown in Figure 1a. CRP‐treated macrophage‐conditioned medium (CRP‐MCM) enhanced tumor proliferation in both 786‐O and MAMIYA cells as compared to non‐treated MCM (Figure 1b). CRP had no direct effect on tumor proliferation in either cell line (Supplementary figure 1a). CRP‐induced activation of the signalling pathway was investigated using a phospho‐kinase array; the results showed that CRP‐MCM enhanced STAT3 activation in MAMIYA cells as compared to MCM (Figure 1c). As shown in Figure 1d, CRP‐MCM induced the phosphorylation of both the Ser727 and Tyr705 sites on STAT3 in both cell lines; the CRP‐MCM‐induced phosphorylation of the Tyr705 site was stronger than that of the Ser727 site, thereby suggesting that unknown factors contained in CRP‐MCM enhance STAT3 activation and tumor proliferation.

Next, we attempted to identify the unknown factors contained in CRP‐MCM that enhance STAT3 activation in the cell lines by using a cytokine array. As shown in Figure 1e, the secretion of CCL2, CCL3/4, CCL5, CXCL1, ICAM1, IL‐1ra, IL‐6 and IL‐21 was increased in CRP‐MCM; these factors were identified as candidate factors inducing STAT3 activation. Among these, IL‐6 is a well‐known inducer of STAT3 activation. An enhanced effect of CRP on both the expression of IL‐6 mRNA and the production of IL‐6 was also observed in human monocyte‐derived macrophages (HMDMs) (Figure 1f and g), whereas CRP had no direct effect on STAT3 activation in ccRCC cell lines (Supplementary figure 1b). Enhanced activation of STAT3 by CRP‐MCM in these cell lines was suppressed by an anti‐IL6R neutralising antibody (Figure 1h).

### CRP induces IL‐6 production via CRP/CD64 signalling pathway in macrophages

**Figure 2:**
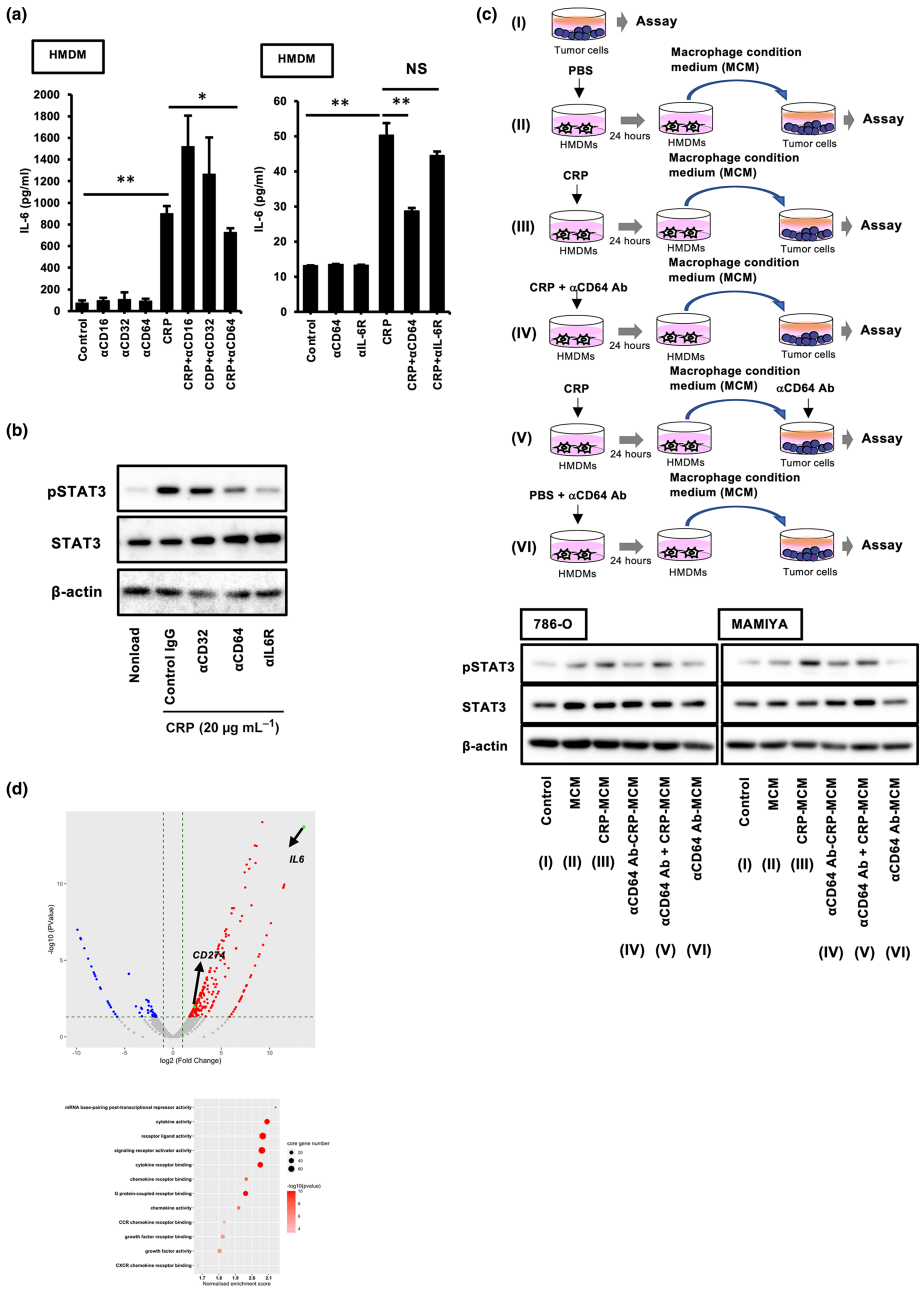
CRP induces the production of IL‐6 via the CRP/CD64 signalling pathway in macrophages. **(a)** The IL‐6 secretion from HMDMs stimulated with CRP (20 μg mL^−1^) during incubation with anti‐CD16, CD32, CD64 and IL‐6R neutralising antibodies for 24 h was measured by ELISA (*n* = 4). Experiments were repeated three times with almost identical results. The Kruskal–Wallis test was performed. *P < 0.01, **P < 0.001. **(b)** The phosphorylated STAT3 expression in HMDMs after treatment with CRP (20 μg mL^−1^) during incubation with anti‐CD64 and IL‐6R neutralising antibodies for 24 h was measured by western blotting. Experiments were repeated three times with almost identical results. **(c)** The phosphorylated STAT3 expression in ccRCC cell lines (786‐O and MAMIYA) after treatment with MCM or CRP‐MCM during incubation with anti‐CD64 neutralising antibody (5 μg mL^−1^) for 24 h was measured by western blotting. Experiments were repeated three times with almost identical results. **(d)** The volcano plots of the gene expression in CRP‐treated HMDMs as measured by RNA‐seq.

To identify the receptors that could be involved in IL‐6 secretion from CRP‐stimulated macrophages, neutralising antibodies were used during the co‐culturing of macrophages with CRP. As shown in Figure 2a, the anti‐CD64 neutralising antibody significantly suppressed CRP‐induced IL‐6 secretion from macrophages, whereas anti‐CD16 and anti‐CD32 neutralising antibodies had no effect; this suggested that the CRP/CD64 axis is involved in CRP‐induced IL‐6 secretion. Anti‐CD64 neutralising antibodies also inhibited STAT3 activation in CRP‐stimulated macrophages (Figure 2b). In addition, CPR and anti‐CD64 neutralising antibody co‐treated MCM (CRP and aCD64 Ab‐MCM) had no effect on STAT3 activation in RCC cell lines (Figure 2c), thereby indicating that IL‐6 induced via CRP/CD64 signalling is involved in STAT3 activation in macrophages. Furthermore, RNA‐seq analysis revealed increased mRNA expression of cytokines, signalling receptors, chemokines and G protein‐coupled receptors in CRP‐stimulated macrophages (Figure 2d). Interestingly, CRP stimulation enhanced CD274 (PD‐L1) expression in macrophages (Figure 2d). Therefore, we examined the effects of CRP on PD‐L1 expression in macrophages.

### CRP induces the expression of PD‐L1 on macrophages via STAT‐1 activation

**Figure 3:**
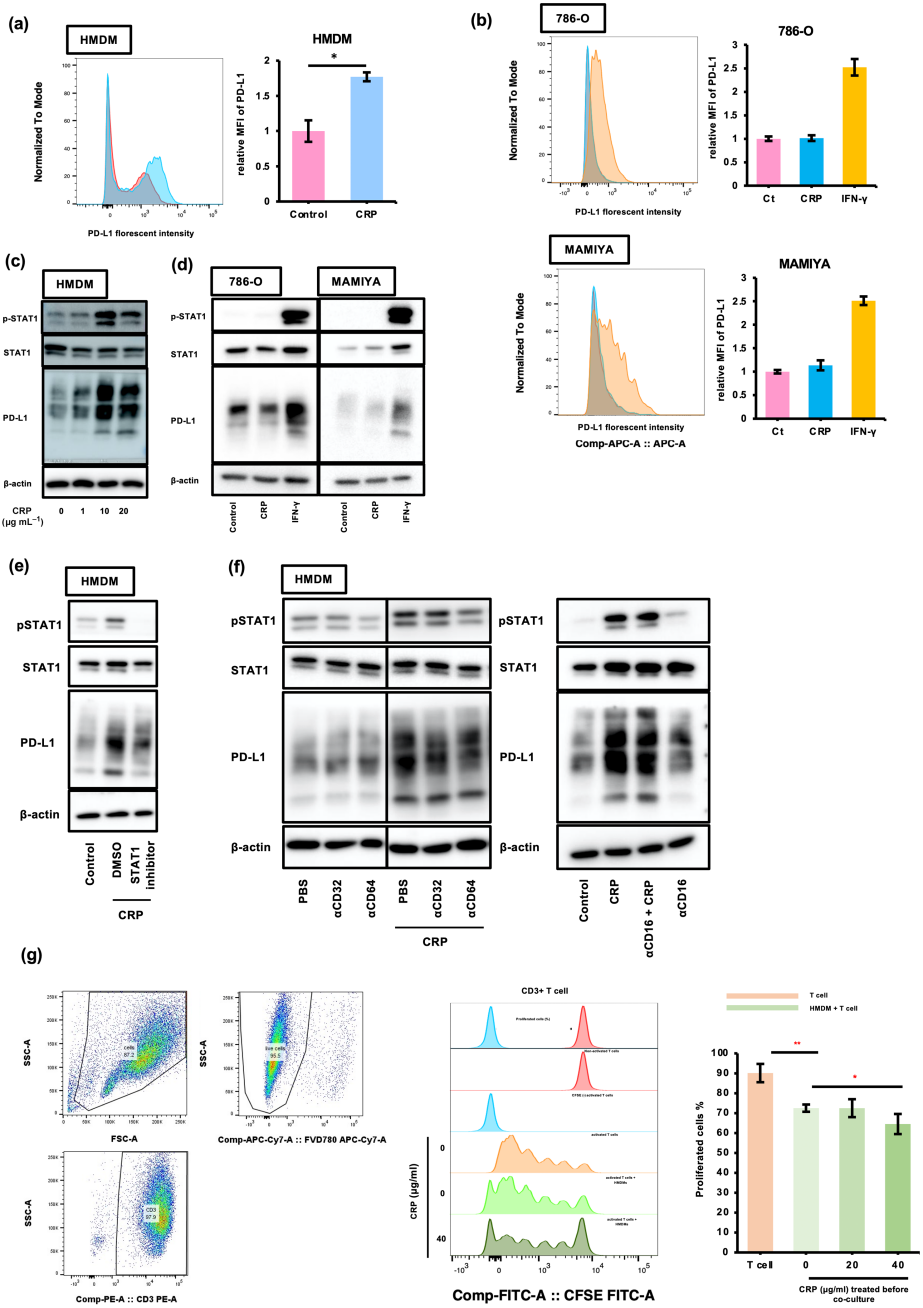
CRP induces the expression of PD‐L1 on macrophages via STAT‐1 activation. **(a)** The PD‐L1 expression in HMDMs after treatment with CRP (20 μg mL^−1^) for 24 h was measured by flow cytometry (*n* = 3). Experiments were repeated three times with almost identical results. The Mann–Whitney *U*‐test was performed. *P < 0.01. **(b)** The PD‐L1 expression in ccRCC cell lines (786‐O and MAMIYA) after treatment with CRP or IFN‐γ for 24 h was measured by flow cytometry (*n* = 3). Experiments were repeated three times with almost identical results. The Kruskal–Wallis test was performed. *P < 0.01. **(c)** The expression of phosphorylated STAT1, STAT1 and PD‐L1 in HMDMs after treatment with the indicated concentration of CRP for 24 h was measured by western blotting. Experiments were repeated three times with almost identical results. **(d)** The expression of phosphorylated STAT1, STAT1 and PD‐L1 in ccRCC cell lines after treatment with CRP or IFN‐γ for 24 h was measured by western blotting. Experiments were repeated three times with almost identical results. **(e)** The expression of phosphorylated STAT1, STAT1 and PD‐L1 in HMDMs after treatment with CRP (20 μg mL^−1^) during incubation with fludarabine, a STAT‐1 inhibitor, for 24 h was measured by western blotting. Experiments were repeated three times with almost identical results. **(f)** The expression of phosphorylated STAT1, STAT1 and PD‐L1 in HMDMs after treatment with CRP (20 μg ml^−1^) during incubation with anti‐CD16, CD32, CD64 and IL‐6R neutralising antibodies (5 μg mL^−1^) for 24 h was measured by western blotting. Experiments were repeated three times with almost identical results. **(g)** CD3^+^ T cells were co‐cultured with HMDMs during incubation with CRP for 24 h, and then stimulated with CD3 and CD28 antibodies for 24 h. The proliferation of activated pan CD3^+^ T cells was measured by flow cytometry (*n* = 3). Experiments were repeated three times with almost identical results. The Kruskal–Wallis test was performed. *P < 0.01, **P < 0.001.

As shown in Figure 3a, CRP induced PD‐L1 expression in HMDMs but had no effect on PD‐L1 expression in tumor cell lines (Figure 3b). IFN‐induced PD‐L1 expression is regulated by the STAT1 signalling cascade in both macrophages and tumor cells.^15^, ^16^ Next, we investigated the effect of CRP on STAT1 activation in macrophages. We found that CRP induced STAT1 activation in HMDMs (Figure 3c) but had no effect in ccRCC cell lines (Figure 3d). Furthermore, CRP‐induced PD‐L1 expression in HMDMs was suppressed by fludarabine, a well‐known STAT1 inhibitor (Figure 3e), suggesting that CRP induced the expression of PD‐L1 in macrophages via STAT1 activation. Anti‐CD64 neutralising antibodies also suppressed CRP‐induced STAT‐1 activation and PD‐L1 expression in HMDMs (Figure 3f), indicating that CRP induced the expression of PD‐L1 via the CRP/CD64/STAT‐1 signalling pathway in macrophages. In addition, CRP‐treated HMDMs inhibited T‐cell proliferation induced by the CD3/CD28 agonist (Figure 3g), demonstrating that CRP was involved in the inactivation of cytotoxic T cells by inducing the expression of PD‐L1 in macrophages.

### The potential contribution of CRP/CD64 signal for ccRCC progression

**Figure 4:**
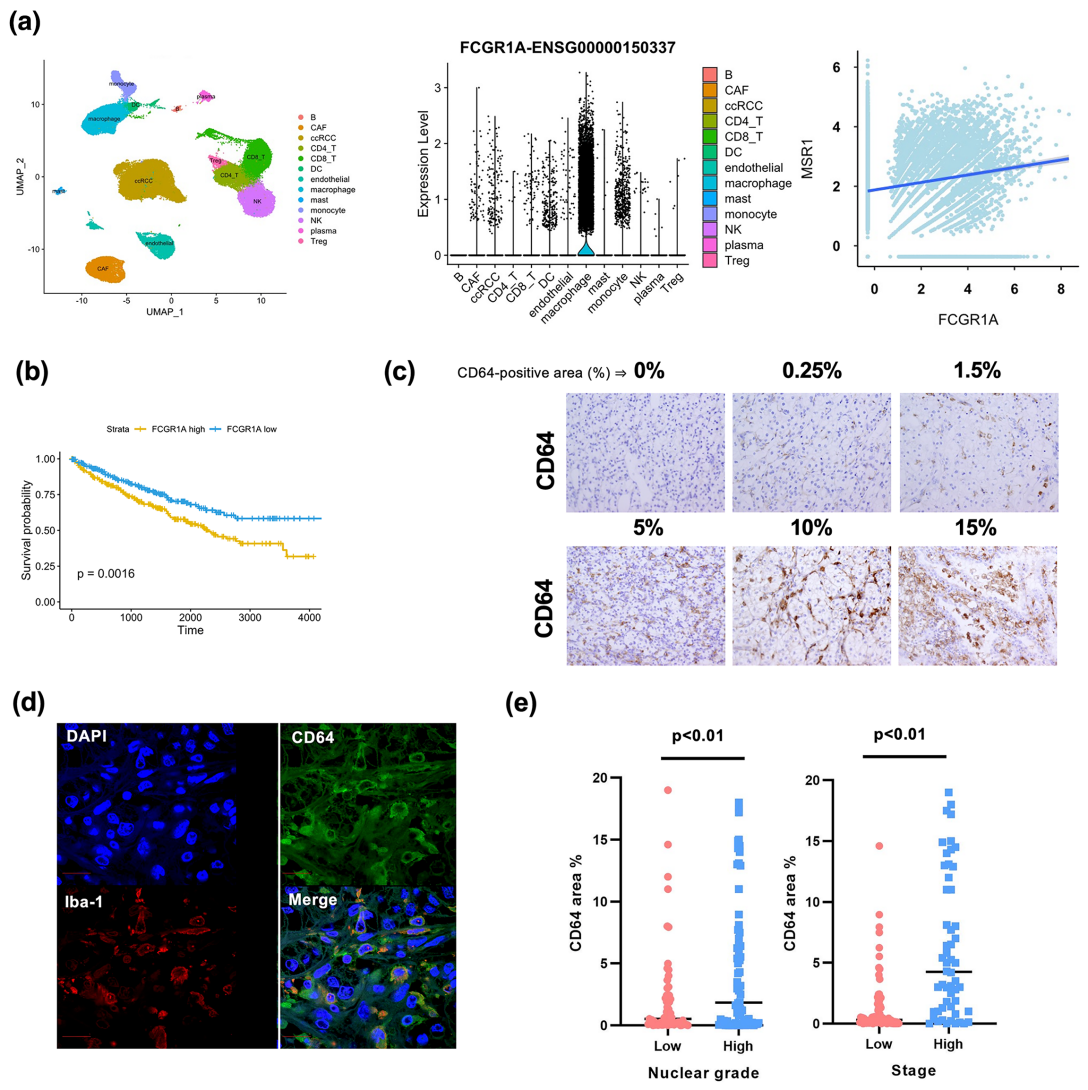
Contribution of CRP/CD64 interaction to the progression of ccRCC. **(a)** The UMAP plots represents 13 cell types from 19 ccRCC tissue samples (left). The expression of FCGR1A (CD64) across these cell types is summarised using violin plots (central). The expression of FCGR1A (CD64) and MSR1 (CD204) is represented by dot plots (right). **(b)** Kaplan–Meier survival curves of ccRCC cases (KIRC cases from TCGA database). The cases were divided into two groups based on the FCGR1A (CD64) expression (FPKM), with the median used as the cut‐off value. **(c)** The CD64 expression in ccRCC tumor tissues (*n* = 133) was detected by IHC. **(d)** Immunofluorescence staining of CD64 (green), Iba‐1 (red) and DAPI (blue) in CRP‐positive ccRCC tissues (*n* = 4). **(e)** Dot plots of the relationship between the percentage of the CD64^+^ area and the Fuhrman nuclear grade/stage in ccRCC cases. The Mann–Whitney *U*‐test was performed.

Next, we used a public data set of the single RNA sequencing (scRNA‐seq) results from 19 ccRCC tissue samples (GSE207493)^17^ to identify cell types expressing CD64 (FCGR1A) in ccRCC tissues. Macrophages expressed high levels of CD64 (FCGR1A) (Figure 4a); a weak correlation between CD64 (FCGR1A) and CD204 (MSR1), a macrophage marker, was detected in ccRCC tissues (Spearman's *R* = 0.157, *P* < 0.01) (Figure 4a). In addition, the summarised and analysed bulk RNA sequencing data from The Cancer Genome Atlas (TCGA) Program of the Human Protein Atlas (https://www.proteinatlas.org/ENSG00000150337‐FCGR1A) showed that ccRCC patients with high CD64 (FCGR1A) expression levels had worse prognoses (Figure 4b). Therefore, we conducted an immunohistochemical analysis of CD64 expression in 128 ccRCC tumor tissues. Patient characteristics are summarised in Table 1. The expression of CD64 was observed to be heterogeneous in ccRCC tumor tissues; CD64 staining was assessed by measuring the CD64^+^ area in ccRCC tumor tissues (Figure 4c). Double‐IHC revealed the expression of CD64 in the macrophages of ccRCC tumor tissues (Figure 4d and Supplementary figure 2). The CD64^+^ area was larger in cases with high nuclear grades (Grades 3 and 4) and stages (Stages III and IV) than in cases with low nuclear grades (Grades 1 and 2) and high stages (Stages I and II) (Figure 4e).

**Table 1:** Patient characteristics

Characteristic	*n* (total = 128)
Sex	
Female	36
Male	92
Age, years	
Median	68
Range	27–85
Histologic subtype	
Clear cell renal cell carcinoma	128
Stage	
1–2	77
3–4	51
Grade	
1–2	62
3–4	66
Preoperative CRP	
Median	0.14
Range	0–16.9

### Increased serum CRP was significantly associated with CD64 expression in tumor tissues and CRP‐positivity in tumor cells

**Figure 5:**
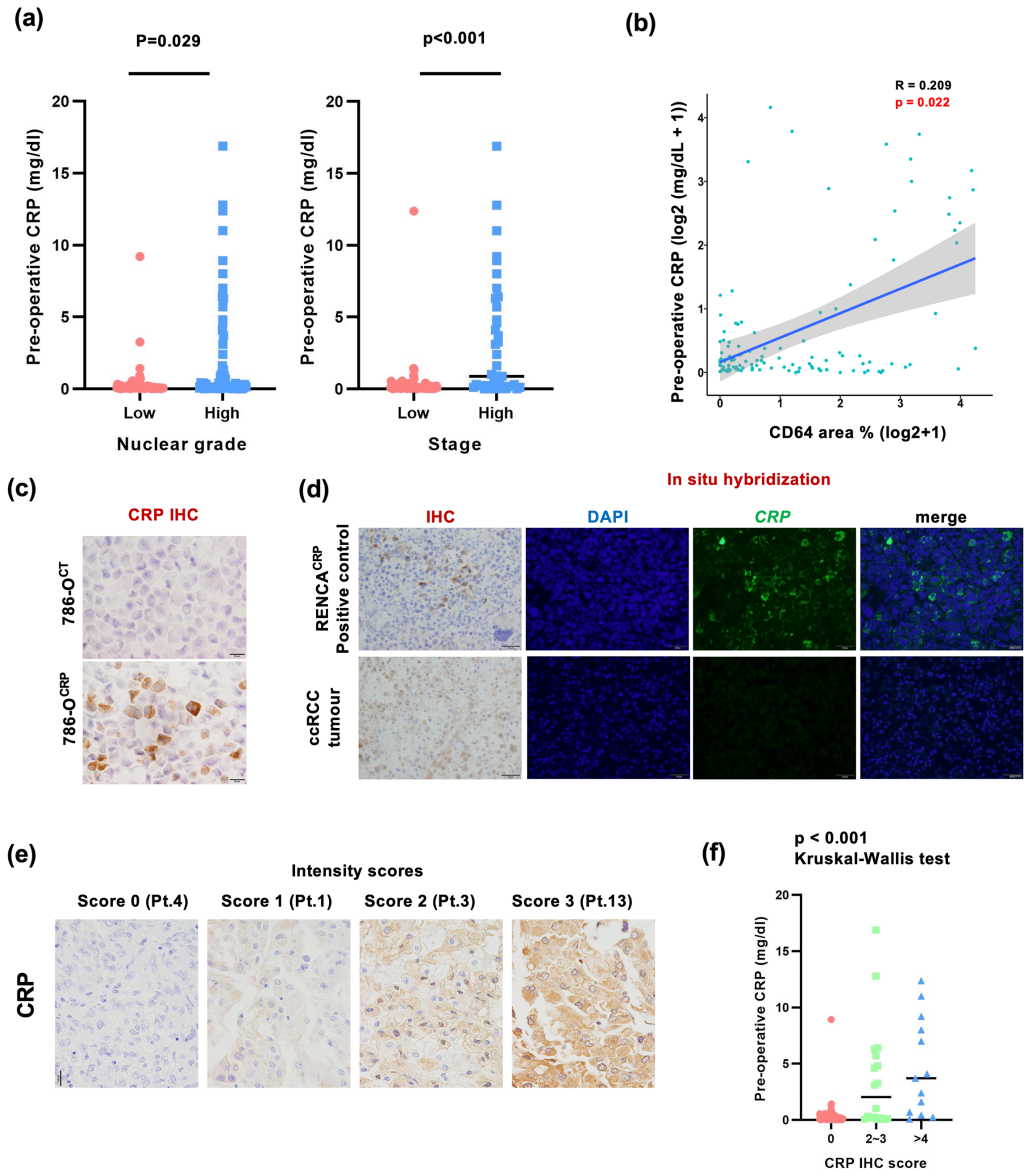
CRP is involved in ccRCC progression via CRP/CD64 interaction in macrophages. **(a)** Dot plots of the relationship between the preoperative serum concentration of CRP in ccRCC cases and Fuhrman nuclear grade/stage in ccRCC tissues (*n* = 133). The Mann–Whitney *U*‐test was performed. **(b)** Dot plots of the relationship between the percentage of the CD64^+^ area and the preoperative serum CRP concentration. **(c)** IHC of CRP in 786‐O^CT^ and 786‐O^CRP^. **(d)** ISH and IHC of CRP in CRP‐positive ccRCC tissue and RENCA^CRP^ cells. **(e)** The intensity score of CRP‐positive cases for IHC in ccRCC tissues. **(f)** Dot plots of the relationship between the CRP IHC score and the preoperative serum CRP concentration (*n* = 119). The Kruskal–Wallis test was performed.

We tested the relationship between serum CRP levels and CD64 expression in cancer tissues. Serum CRP levels significantly correlated with nuclear grade, stage and CD64 expression (Figure 5a); there was a significant positive association between serum CRP levels and CD64 expression (Figure 5b).

Because IHC for CRP is available in paraffin sections, it was additionally performed in ccRCC tissue sections. The specificity of the anti‐CRP antibody was examined using a CRP‐gene‐transfected cancer cell line (Figure 5c). Positive CRP signals were observed in 31% of the ccRCC cases; however, CRP mRNA was not detected using ISH (Figure 5d). CRP positivity was assessed by a scoring system using the sum of the CRP area percentage and staining intensity scores, as described in the Methods section (Figure 5e). There was a significant correlation between the CRP IHC score in cancer tissues and its serum level (Figure 5f), suggesting that the interaction of CRP with CD64‐expressing macrophages in the tumor microenvironment may play a critical role in ccRCC tumor progression.

## Discussion

Immune checkpoint inhibitors (ICI) have been used to treat various cancers owing to their promising effects; however, not all patients respond favorably to ICI therapy. Therefore, identifying patients who are sensitive to ICI therapy and the monitoring of their responses are essential. Several recent clinical studies have focussed on CRP level as a prognostic factor in ICI therapy for cancer. In non‐small‐cell lung carcinoma (NSCLC), melanoma, urothelial carcinoma and RCC, a meta‐analysis of 13 cohorts treated with ICIs revealed that high baseline CRP levels were associated with low overall survival (OS) and progression‐free survival (PFS) in NSCLC, melanoma, urothelial carcinoma and RCC.^18^ In patients with metastatic RCC who received immunotherapy, CRP was also positively associated with worse OS and PFS, and more accurately correlated with survival prognosis than factors in the International Metastatic RCC Database Consortium (IMDC),^19^ suggesting that CRP is associated with a decreased response to ICI therapy. Furthermore, high CRP levels have been reported to induce an immunosuppressive environment by inhibiting T‐cell proliferation and downregulating co‐stimulatory molecules on dendric cells (DCs) in melanoma,^20^ indicating that CRP blockade is a therapeutic strategy to enhance ICI therapy in cancer.

In the present study, we identified CRP as an acute‐phase protein that binds to CD64 and induces PD‐L1 expression and IL‐6 production in macrophages (Figures 2 and 3). Since PD‐L1 is an immune checkpoint molecule that inhibits the activation of T cells by binding to PD‐1 expressed on CD8^+^ killer T cells,^21^ anti‐PD‐L1 antibody therapy has been successfully used as an immunotherapy agent for various cancers, including melanoma and NSCLC.^22^, ^23^ A recent study revealed that both tumor cells and macrophages express PD‐L1 and significantly contribute to tumor immunosuppression in tumor tissues.^24^ CRP‐treated HMDMs inhibited the proliferation of activated T cells (Figure 3g), suggesting that CRP may contribute to immunosuppression in tumor tissues by inducing the expression of PD‐L1 in macrophages. IL‐6 secreted from CRP‐stimulated HMDMs also induced STAT3 activation in both HMDMs and tumor cells (Figure 1). The crosstalk via STAT3 between immune and tumor cells is known to promote tumor progression by enhancing tumor cell proliferation and survival as well as immunosuppression.^25^ In addition, there was a significant correlation between CRP and IL‐6 concentrations in the serum of RCC patients,^26^ suggesting that IL‐6 secreted from RCC tissues stimulates the secretion of CRP from the liver, which in turn accelerates IL‐6 expression in macrophages. Therefore, CRP may also contribute to tumor progression by inducing the production of IL‐6 by macrophages, followed by STAT3 activation in tumor tissues. This suggests that in the clinical setting, monitoring serum CRP may be useful for predicting the prognosis of ccRCC, and that maintaining a low serum CRP level may be beneficial for anticancer therapy.

**Figure 6:**
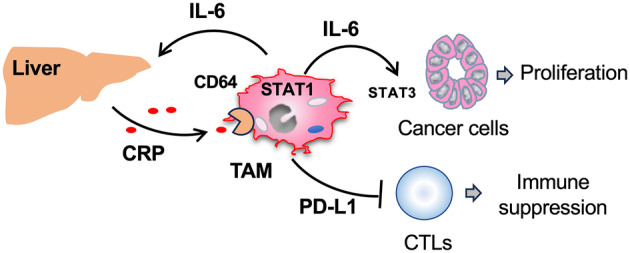
The CRP/CD64 axis contributes to renal cancer progression by inducing protumor activation of tumor‐associated macrophages.

CD64 (Fcγ receptor I), expressed in macrophages, monocytes, granulocytes and DCs, is involved in the induction of phagocytosis and pro‐inflammatory cytokine/chemokine secretion.^27^ Along with CD16 and CD32, CD64 is a known receptor for CRP on macrophages.^28^ Marjon KD et al. previously reported that CRP suppresses immune thrombocytopenic purpura (ITP), an immune complex disease, in a mouse model by inducing suppressive macrophages through binding to CD64.^29^ This suggests that CRP may also promote PD‐L1 expression in macrophages via the CRP/CD64 axis, potentially contributing to ITP suppression. In antibody medicine for the treatment of metastatic melanoma, the recognition of the Fc portion of IgG by macrophage CD64 is known to be essential for the elimination of antibody‐bound tumor cells in the tumor microenvironment.^30^ However, our study found that CD64 was largely expressed by macrophages in ccRCC tissues and that the expression of CD64 was associated with a poor prognosis (Figure 4). CRP induced the expression of both IL‐6 and PD‐L1 in HMDMs by binding to CD64, but not to CD16 or CD32 (Figures 2 and 3), indicating that the interaction of CRP with macrophage CD64 may promote the progression of ccRCC by triggering the protumor function of macrophages (Figure 6).

Several studies have reported the role of CRP as a predictor of ICI efficacy in RCC.^31^, ^32^, ^33^ These studies showed that a high serum CRP level is associated with a poor response to ICI therapy.^31^, ^32^, ^33^ It has also been reported that NSCLC patients with a high PD‐L1 TPS level respond well to ICIs. However, patients with both high PD‐L1 TPS and serum CRP levels responded worse than those with high PD‐L1 TPS but low serum CRP.^34^ This suggests that ICI responsiveness is more likely to be compromised by high CRP levels than enhanced by CRP‐induced PD‐L1 expression. We speculate that this phenomenon occurs not only because CRP enhances the expression of PD‐L1 in TAMs but also because it contributes to the production of IL‐6 in TAMs and the activation of STAT3 in tumor tissues, thereby accelerating the proliferation of ccRCC tumor cells and reducing the effectiveness of ICI treatment. Therefore, blocking the CRP/CD64 axis, which can suppress both PD‐L1 expression and IL‐6 secretion, may be highly effective in patients with high serum CRP levels. However, this remains as a subject for future investigation.

The effect of CRP on tumor progression needs to be examined in *in vivo* models, such as the tumor‐bearing mouse model; however, this remains challenging. Unlike amyloid A (SAA) and α‐acid glycoprotein (AGP), CRP is not considered as an acute‐phase protein in mouse models because it increases only two‐ to threefold under inflammatory conditions.^35^, ^36^, ^37^ In addition, although CRP‐deficient mice^37^, ^38^ have been frequently used in atherosclerosis and bacterial infection studies, neither the expression of PD‐L1 nor the activation of STAT3 was observed in mice bone marrow‐derived macrophages in our study (data not shown), suggesting that CRP is not suitable for tumor‐bearing mouse models. However, human CRP induced both the expression of PD‐L1 and the activation of STAT3 in mouse bone marrow‐derived macrophages, similar to HMDMs (data not shown); therefore, validated animal models are needed to study the biological functions of CRP in various diseases, including cancer.

Our study indicates that elevated serum CRP levels may contribute to CRP levels in ccRCC tissues and that the CRP/CD64 axis induces PD‐L1 and IL6 expression in TAMs, thereby contributing to the progression of ccRCC. Therefore, low serum CRP levels in ccRCC patients undergoing anticancer therapy may enhance the efficacy of therapy, wherein CD64 may be a novel therapeutic target for ccRCC.

## Methods

### Cells and cell culture

Human ccRCC cell lines (786‐O and MAMIYA) were purchased from the RIKEN Cell Bank (Tsukuba, Japan) and cultured in RPMI1640 medium (FUJIFILM Wako Pure Chemical Corporation, Tokyo, Japan) supplemented with 10% foetal bovine serum (FBS; Gibco, Grand Island, NY, USA) and 1% penicillin–streptomycin solution (FUJIFILM Wako Pure Chemical Corporation).

Peripheral blood mononuclear cells (PBMCs) were isolated from human peripheral blood samples obtained from healthy donors who consented to use the samples. All protocols involving human material were approved by the Kumamoto University Review Board (No. 486). For macrophage differentiation, adherent mononuclear cells were cultured in Corning® Primaria™ tissue culture dishes (Corning Inc., Corning, NY, USA) with 10 ng mL^−1^ human recombinant M‐CSF (FUJIFILM Wako Pure Chemical Corporation, Tokyo, Japan) for 7 days. Differentiated HMDMs were used for the experiments. Differentiated HMDMs were cultured in low‐glucose DMEM (FUJIFILM Wako Pure Chemical Corporation, Tokyo, Japan) supplemented with 2% FBS and 1% penicillin–streptomycin solution.

Human T cells were isolated from PBMCs. PBMC suspensions were collected and subjected to a pan T‐cell isolation kit (130‐096‐535, Miltenyi Biotec B.V. & Co. KG, Germany). Non‐T‐cell components were bound to antibodies and selected using microbeads, while non‐selected cells served as T cells. Human T cells were cultured in RPMI1640 medium supplied with 10% foetal bovine serum and 1% penicillin–streptomycin.

### CRP‐treated macrophage‐conditioned medium (CRP‐MCM)

Human monocyte‐derived macrophages (2 × 10^4^/well) in a 24‐well plate were cultured with or without human CRP (20 μg mL^−1^) for 24 h. Supernatants were harvested and centrifuged to remove floating cells and debris. The supernatants were then diluted with tumor cell medium (1:1). The diluted supernatants served as the MCM and CRP‐MCM for subsequent experiments.

### Reagents and chemicals

The antibodies and reagents used in this study were as follows: anti‐human CD16 antibody (mouse IgG1, κ, clone: 3G8, 302001, BioLegend, CA, USA), anti‐human CD32 antibody (mouse IgG2b, κ, clone: IV.3, 60012, STEMCELL™ Technologies Inc., Canada), anti‐human CD64 antibody (mouse IgG1, κ, clone:10.1, 305002, BioLegend, CA, USA), mouse IgG2b, κ isotype antibody (clone: MPC‐11, 400301, BioLegend, CA, USA), mouse IgG1, κ isotype antibody (clone: MOPC‐21, 400102, BioLegend, CA, USA), anti‐human CD3 antibody (mouse IgG2a, κ, clone: OKT3, 16‐0037‐85, Invitrogen, CA, USA), anti‐human CD28 antibody (mouse IgG1, κ, clone: CD28.2, 302923, BioLegend, CA, USA), STAT3 inhibitor WP1066 (sc‐203282, ChemCruz, Netherlands), fludarabine, a STAT1 inhibitor (Cayman Chemical, USA), humanised monoclonal antibody against the interleukin‐6 receptor (IL‐6R, Actemra, Tocilizumab, jan code: 4987136118600, Chugai Pharmaceutical Co. Ltd, Japan) and FcR Blocking Reagent, human (130‐059‐901, Miltenyi Biotec B.V. & Co. KG, Germany). Human Kidney Tissue MicroArray slides (NBP2‐30220) were purchased from Novus Biologicals (CO, USA). The HilyMax gene transfection reagent was purchased from Dojindo Molecular Technologies, Inc. (Japan). The expression plasmid cDNA of the CRP (NM_000567) human‐tagged ORF clone was purchased from OriGene Technologies, Inc.

### Flow cytometry for PD‐L1 in HMDMs and ccRCC cells

For HMDMs, cells were seeded in a 12‐well plate at a density of 20 × 10^4^ per well, and treated with 20 μg mL^−1^ human CRP for 24 h. The 786‐O or MAMIYA cells seeded in a 12‐well plate at 70% confluency were treated with 20 μg mL^−1^ human CRP for 24 h. The cells were harvested and stained with APC‐anti‐human CD274 (B7‐H1, PD‐L1) mouse monoclonal antibody (IgG2b, κ, clone: 29E.2A3, 329707, BioLegend, CA, USA) for 15 min at 4°C. The cells were washed twice with FACs buffer (PBS containing 2 mM EDTA and 0.5% FBS, pH 7.2) and subjected to flow cytometry. The geometric mean value of PD‐L1 was used to calculate the average fluorescence intensity.

### Flow cytometry for activated T‐cell activation

A 96‐well plate was coated with anti‐human CD3 and anti‐human CD28 antibodies, and incubated overnight. HMDMs were seeded at a density of 2 × 10^4^ per well for 24 h. HMDMs were then treated with different concentrations of human CRP for 24 h. The supernatants were removed; the HMDMs were then washed with PBS. Human T cells (2 × 10^5^ per well) were labelled with CFSE (2 μM; Dojin Chemical, Kumamoto, Japan) for 30 min and washed with PBS. CFSE‐labelled human T cells were added to the plate and co‐cultured with CRP‐treated HMDMs for 24 h. The suspended cells were harvested and stained with FVD780 and FcR‐blocking reagents. The cells were then stained with a PE‐anti‐human CD3 mouse monoclonal antibody (IgG2a, κ, clone: HIT3a, 300307, BioLegend, CA, USA) and washed with FAC buffer. The cells were then subjected to flow cytometry. The fluorescence compensation was adjusted, wherein FVD780^−^ CD3^+^ cells were gated as human T cells. The intensity of FITC fluorescence was treated as the intensity of CFSE.

### Cell proliferation assay

The 786‐O or MAMIYA cells were cultured in a 96‐well plate (500 per well) overnight. Then, CRP was added at different concentrations via MCM and CRP‐MCM, and cultured for 24 h. Cell viability was determined using a Cell Counting Kit‐8 assay (WST‐8 assay, Dojin Chemical, Kumamoto, Japan) according to the manufacturer's protocol.

### Human phospho‐kinase array analysis

The MAMIYA cells were treated with MCM or CRP‐MCM for 24 h. Lysates of MAMIYA cells were harvested using the Proteome Profiler™ Array Human Phospho‐Kinase Array Kit (ARY003B, R&D Systems, Inc., MN, USA). Experiments were performed according to the manufacturer's instructions.

### Human cytokine array analysis

The MCM and CRP‐MCM were subjected to human cytokine array analysis using the Proteome Profiler Array Human XL Cytokine Array Kit (ARY022; R&D Systems, Inc., Minneapolis, MN, USA). Experiments were performed according to the manufacturer's instructions.

### Western blotting analysis

Cell lysates were harvested using the NP‐40 lysis buffer. The proteins were quantified using the BCA method. Each prepared cell lysate sample, containing 10 μg of protein, was used for SDS‐polyacrylamide electrophoresis. Proteins were transferred to PVDF membranes (Millipore, Bedford, MA, USA). The membranes were then blocked with 1% skim milk (FUJIFILM Wako Pure Chemical Corporation, Tokyo, Japan) and probed with primary antibodies. The primary antibodies used in this study were as follows: anti‐phosphorylated STAT3 (Tyr705) antibody (clone: D3A7; #9145, Cell Signaling Technology), anti‐phosphorylated STAT3 (Ser727) antibody (polyclonal, #9134, Cell Signaling Technology), anti‐STAT3 antibody (clone: 124H6, #9139, Cell Signaling Technology), anti‐STAT3 antibody (clone:124H6, #9139, Cell Signaling Technology), anti‐phosphorylated STAT1 (Tyr701) antibody (clone: 58D6, #9167, Cell Signaling Technology), anti‐STAT1 antibody (clone: 42H3; #9175, Cell Signaling Technology), anti‐PD‐L1 antibody (clone: E1L3N; #13684, Cell Signaling Technology) and anti‐β‐actin antibody (clone: C4, sc‐47 778, Cell Signaling Technology). The membranes were then probed with primary and secondary antibodies. The secondary antibodies used in this study were horseradish peroxidase‐conjugated goat anti‐mouse IgG (62‐6520, ThermoFisher Scientific) and horseradish peroxidase‐conjugated goat anti‐rabbit IgG (ab6721, Abcam). The chemiluminescent signal was developed using a WSE‐7120L EzWestLumi plus (2332637, ATTO Corporation, Japan) and detected using an Amersham Imager 680 (Global Life Sciences Solutions LLC, MA, USA).

### Real‐time quantitative reverse transcription PCR (qRT‐PCR)

HMDMs were harvested using RNAiso Plus (TAKARA, Japan); the total RNA of each sample was then isolated. Total RNA, with the DNA being removed, was reverse‐transcribed into cDNA using a PrimeScript RT Reagent Kit (TAKARA, Japan). The cDNA from each sample was adjusted to the same concentration and used for qRT‐PCR. TaqMan polymerase with SYBR Green fluorescence (TAKARA, Otsu, Japan) was used for qRT‐PCR; the signal was detected using an ABI PRISM 7300 Sequence Detector (Applied Biosystems, Foster City, CA, USA). The sequences of the primers for human IL‐6 were as follows: Forward (5′–3′), ATGTGTGAAAGCAGCAAAGAGG; reverse (5′–3′), GTGATGATTTTCACCAGGCAAG.

### Enzyme‐linked immunosorbent assay (ELISA)

Human monocyte‐derived macrophage supernatants were harvested and centrifuged to remove floating cells and cell debris. The quantity of IL‐6 in the supernatants was measured using the IL‐6 Human Uncoated ELISA Kit (88‐7066‐88; Invitrogen).

### Bulk RNA sequencing of HMDMs

Cells were treated with CRP; total RNA was isolated using RNAiso Plus (Takara Bio). Total RNA was prepared by TRIzol extraction (Thermo Fisher), wherein the quality was confirmed using a BioAnalyzer 2100 (RNA integrity number > 9). For the RNA‐seq data analysis, the resulting reads were aligned to the human genome (UCSC hg19) via STAR ver.2.6.0a, after trimming to remove adapter sequences and low‐quality ends via Trim Galore! v0.5.0 (cutadapt v1.16). Gene expression levels were measured in terms of transcripts per million, as determined using RSEM v1.3.1. Raw gene counts were used to identify differentially expressed genes (DEGs). The R package *edgeR* was used to identify DEGs. A square‐root‐dispersion (BCV) of 0.4 was adapted for the Fisher's exact test. DEGs were genes with a fold change > 2 or < 0.5 and a *P*‐value < 0.05. All genes were used for gene set enrichment analysis (GESA) for GO biological processes using the R packages *org.Hs.eg.db* and *clusterProfiler*. The ranking metric of the genes was as follows:

$$\text{ranking value}=-\mathrm{sgn}\left\{\log_{10}\text{(fold change)}\right\}･\log_{10}\left(P-\text{value}\right).$$

### The transfection of human CRP cDNA into 768‐O cells

The expression plasmid cDNA of *CRP* was reconstituted in DW according to the manufacturer's protocol. The 786‐O cells were cultured in a six‐well plate at 50% confluency overnight; CRP cDNA was then transfected into the cells using HilyMax reagent according to the manufacturer's protocol. The cells were then cultured for 24 h. The supernatants were then removed; the medium containing G‐418 was then added. RT‐PCR was used to test whether G‐418‐selected cells expressed *CRP*. Cells expressing the *CRP* gene were used to generate single‐cell clones. The cell suspension was diluted to 1 cell per 100 μL; the cells were then seeded into 96‐well plates (1 cell per well). Proliferating cells in each well were examined for the expression of *CRP*. Clones of 786‐O cells expressing high levels of *CRP* and clones not expressing *CRP* were used to validate CRP immunohistochemical staining and *in situ* hybridisation.

### Immunochemistry (IHC)

Paraffin‐embedded ccRCC tumor samples were obtained from 133 patients. Ninety‐five cases were from Kumamoto University Hospital, while another 38 were from Toranomon Hospital. Patient characteristics, including sex, subject demographics and histological subtypes, are shown in Table 1. The study design was approved by the Institutional Review Boards of Kumamoto University (#2059) and Tranomon Hospital (#945), in accordance with the guidelines for Good Clinical Practice and the Declaration of Helsinki.

A thin section (3 μm) from each sample was prepared and used for immunochemical staining of CRP. Heat‐induced antigen retrieval was then performed on these sections. The sections were incubated in 1% hydrogen peroxide to block endogenous peroxidase, blocked with 5% goat serum and incubated overnight with an anti‐CRP antibody (ab31156, Abcam). The sections were washed with PBS and incubated with an HRP‐conjugated secondary antibody (Nichirei, Tokyo, Japan). Visualisation was performed using 3, 3′‐diaminobenzidine tetrahydrochloride (DAB, code425011 Nichirei). A scoring system was adapted to assess for CRP staining. The total CRP score was calculated as the sum of the staining proportion and intensity scores. The intensity of the CRP staining score was divided into four tiers (0, negative; 1, weak; 2, intermediate; 3, strong). The proportion score was also divided into four tiers (0, none; 1, < 10%; 2, 10–50%; 3, > 50%).

### *In situ* hybridisation (ISH)

*In situ* hybridisation was performed on paraffin sections using the RNAscope® 2.5 HD Duplex Detection Kit (#322500‐USM; Advanced Cell Diagnostics, Newark, CA, USA), according to the manufacturer's instructions.

### Single‐cell RNA sequencing (scRNA‐seq) data analysis

The R package *Seurat* was used to analyse the scRNA‐seq data. scRNA‐seq data from 19 ccRCC cases were first loaded and merged into a single *Seurat* object. The data were then normalised using the ‘*LogNormalize*’ method; the top 2000 highly variable genes were used for the subsequent analysis. The R package *harmony* was used to remove batch effects. The *ElbowPlot* function was used to determine the most informative principal components of the PCA for the *harmony* object; the top 30 components were used for further analysis. The cells were clustered with a resolution of 0.5, using *FindClusters*. Twenty cell clusters were identified; DEGs in different clusters were identified using the FindAllMarker function, *FindAllMarkers*. The cell types in each cluster were identified using markers suggested in the original report.^15^ Thirteen cell types (B cells, CAF, ccRCCs, CD4 T cells, CD8 T cells, DC, endothelial cells, macrophages, mast monocytes, NK cells, plasma cells and Tregs) were identified. The expression of CD64 (FCGR1A) across these cell types is summarised using a violin plot. The expression levels of CD64 (FCGR1A) and CD204 (MSR1) were plotted as dot plots; the correlation was analysed using Spearman's rank correlation coefficient.

### The analysis of the data from The Human Protein Atlas

Bulk RNA sequencing data of 528 ccRCC (KIRC) cases from TCGA are summarised in The Human Protein Atlas (https://www.proteinatlas.org/about/download). The median number of Fragments Per Kilobase of exon per million reads (FPKM) of CD64 (FCGR1A) was used as the cut‐off value to divide the cases into two groups. The Kaplan–Meier estimator was used to compare the prognoses of the two groups. The significant differences between each group were examined using the log‐rank test; *P*‐values of < 0.05 were considered to be statistically significant.

### Experimental replication

All experiments were performed with two or three independent replicates.

### Statistical analyses

Unless specifically described, data are presented as the mean ± SD. All experiments were performed with two or three independent replicates. The EZR or Prism software was used to perform statistical analyses. The Student's *t*‐test was used to compare the differences between two independent Gaussian groups. The Mann–Whitney *U‐*test was used to compare differences between two independent non‐Gaussian groups. For differences in more than two independent groups, the Kruskal–Wallis test was used. *P*‐values of < 0.05 were considered to be statistically significant.

## Author contributions

**Cheng Pan:** Conceptualization; data curation; writing – original draft. **Yukio Fujiwara:** Conceptualization; data curation; funding acquisition; writing – original draft; writing – review and editing. **Hiromu Yano:** Data curation; writing – original draft. **Toshiki Anami:** Data curation. **Yuki Ibe:** Data curation. **Lianbo Li:** Data curation. **Yuji MIura:** Data curation. **Takanobu Motoshima:** Data curation. **Shigeyuki Esumi:** Data curation. **Junji Yatsuda:** Data curation. **Taizo Hibi:** writing – review and editing. **Tomomi Kamba:** writing – review and editing. **Yoshihiro Komohara:** Conceptualization; writing – original draft; writing – review and editing; funding acquisition.

## Conflict of interest

The authors declare no conflict of interest.

## Ethics approval and consent to participate

The study design was approved by the Institutional Review Boards of Kumamoto University (#2059) and Tranomon Hospital (#945), in accordance with the guidelines for Good Clinical Practice and the Declaration of Helsinki. HMDMs and lymphocytes were obtained from healthy donors in accordance with protocols approved by the Kumamoto University Hospital Review Board (No. 1169).

## Funding information

This study was supported by JSPS KAKENHI (grant numbers 20H03459 to YK and 23K06362 to YF).

## Supporting information


Supplementary figure 1
Supplementary figure 2

## Data Availability

All data are available from the corresponding author upon reasonable request.
