# Supplementary material for: The contribution of the CRP/CD64 axis to renal cancer progression by inducing protumor activation of tumor‐associated macrophages
Source: Clin Transl Immunology. 2024 Nov 19;13(11):e70013. doi: 10.1002/cti2.70013 (PMC11574560; doi:10.1002/cti2.70013)
Supplement: Supplementary file 1 — Supplementary figure 1 Supplementary figure 2 [file CTI2-13-e70013-s001.docx]

**
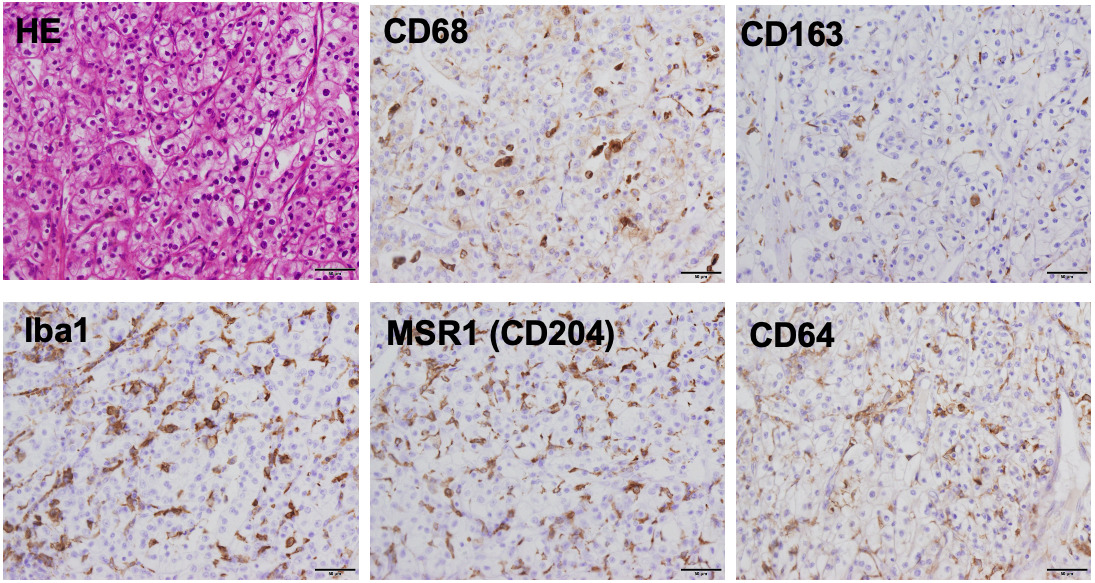
**

**Supplementary figure 2.** Eosin and hematoxylin (HE), CD68, CD163, Iba1, MSR1(CD204), and CD64 IHC in CRP-positive ccRCC tissues.
